# Developing and validating a novel questionnaire to capture bio-psycho-social variables of allergic reactions in the community: the AlleRiC study and the preliminary analyses

**DOI:** 10.1186/2045-7022-5-S3-P1

**Published:** 2015-03-30

**Authors:** Katarzyna Pyrz, Aida Semic-Jusufagic, Christopher Munro, Philip Couch, Clare Mills, Jonathan Hourihane, Audrey Dunn Galvin

**Affiliations:** 1 University College Cork, Cork, Ireland; 2 University of Manchester, Manchester, UK

## Aims

Allergic disease is a growing health risk in the modern world, while its management at professional and patients’ levels is unsatisfactory. There is no register of prevalence and biopsychosocial co-factors of allergic reactions as they occur in real world settings.

The Allergic Reactions in the Community (AlleRiC) study aims to develop and validate an on-line questionnaire to allow real time food allergic reactions to be reported, with scope for an in depth exploration of related real-world factors.

## Methods

Following focus groups and an expert evaluation, an item pool of 81 items was generated. 39 adults from Ireland and UK, diagnosed with food allergy, evaluated a prototype of the AlleRiC online questionnaire. Individual items of the prototype were psychometrically assessed via a novel Evaluative Scale measuring five different facets of the questions.

## Results

Individual items were evaluated positively by participants (60-70% of positive scores on the Evaluative Scale). Preliminary psychometric analyses of the questionnaire’s items showed good construct, convergent and face validity (Cronbach’s Alpha >0.7). With reference to co-factors, reactions occurred most often in a social context (67% of cases), especially in small groups (29% of cases) of family members, relatives and close friends (44% of cases). 77% of companions were aware of the participants’ food allergy diagnosis. Reactions had a psychological impact with 87% of cases indicating a change in emotional affect following a reaction (from positive to negative affect).

## Conclusions

The AlleRiC study demonstrates potential to provide novel findings, which may give new understanding of the real-world, real time factors that give rise to, or prevent, reactions. Such findings will have implications for food allergy management across Europe. As a new psychometric tool: the AlleRiC questionnaire is now undergoing further validation.

